# An Integrated Proteomic and Metabolomic Study on the Chronic Effects of Mercury in *Suaeda salsa* under an Environmentally Relevant Salinity

**DOI:** 10.1371/journal.pone.0064041

**Published:** 2013-05-16

**Authors:** Xiaoli Liu, Huifeng Wu, Chenglong Ji, Lei Wei, Jianmin Zhao, Junbao Yu

**Affiliations:** 1 Key Laboratory of Coastal Zone Environmental Processes, Yantai Institute of Coastal Zone Research (YIC), Chinese Academy of Sciences (CAS); Shandong Provincial Key Laboratory of Coastal Zone Environmental Processes, YICCAS, Yantai Shandong, P. R. China; 2 The Graduate School of Chinese Academy of Sciences, Beijing, P. R. China; The Ohio State University, United States of America

## Abstract

As an environmental contaminant, mercury is of great concern due to its high risk to environmental and human health. The halophyte *Suaeda salsa* is the dominant plant in the intertidal zones of the Yellow River Delta (YRD) where has been contaminated by mercury in some places. This study aimed at evaluating the chronic effects of mercury (Hg^2+^, 20 µg L^−1^) and the influence of an environmentally relevant salinity (NaCl, 500 mM) on mercury-induced effects in *S. salsa*. A total of 43 protein spots with significant changes were identified in response to Hg^2+^, salinity and combined Hg^2+^ and salinity. These proteins can be categorized into diverse functional classes, related to metabolic processes, photosynthesis, stress response, protein fate, energy metabolism, signaling pathways and immunosuppression. Metabolic responses demonstrated that Hg^2+^ could disturb protein and energy metabolisms in *S. salsa* co-exposed with or without salinity. In addition, both antagonistic and synergistic effects between Hg^2+^ and salinity were confirmed by differential levels of proteins (magnesium-chelatase and ribulose-l,5-bisphosphate carboxylase/oxygenase) and metabolites (valine, malonate, asparagine, glycine, fructose and glucose) in *S. salsa*. These findings suggest that a combination of proteomics and metabolomics can provide insightful information of environmental contaminant-induced effects in plants at molecular levels.

## Introduction

Mercury is a non-essential and highly toxic heavy metal to organisms [1]. It can be found in cells bound with thiol-containing proteins, glutathione (GSH) and cysteine. In addition, mercury can cause oxidative stresses by producing reactive oxygen species (ROS) such as hydrogen peroxide, superoxide radical and hydroxyl radical in plant cells and lipid peroxides and consequently damages protein, lipid and DNA [2]. In some intertidal zones of the Yellow River Delta (YRD), mercury is a severe heavy metal contaminant and has posed great risk to the intertidal organisms due to its high toxicity [3]. The mercury concentration has been as high as 66 mg kg^−1^ in the intertidal sediment in some mercury-polluted sites in the YRD [3]. In soils and waters, Hg (II) is the predominant form that can be easily accumulated in plants [1]. Realistically, however, the intertidal zones are of a high salinity which is one of the most common environmental stressors resulting in osmotic pressure, water deficit, ion toxicity and nutrient deficiency in plants [4]. Therefore salinity should be considered in environmental toxicology since it can potentially influence contaminant-induced effects in organisms.

The halophyte *Suaeda salsa* is the pioneer plant in the intertidal zones of the YRD, where salinity is often up to 3% [5]. This plant species is consumed as an edible vegetable by local residents because of its beneficial nutrient components such as vitamins, proteins and antioxidant ingredients [5]. *S. salsa* has also demonstrated the virtues in environmental sciences [6], [7]. For example, it has been applied in phyto-remediation for heavy metals and oil in saline soil [6], [7] and deemed as an environmental bioindicator for heavy metals in the intertidal zones because of its substantial tolerance to environmental salinity compared with animals [8]. In this study, we selected *S. salsa* as the experimental plant to evaluate the influence of environmental salinity on the toxicological effects of environmental relevant mercury in the intertidal zones in the YRD.

Proteomics and metabolomics are two well-established “–omic” techniques in the post-genomic era [9]–[11]. Proteomics is the study of large-scale proteins in an organism encoded by its genome [12]. Not only is proteomics a powerful tool for describing complete proteomes at organelle, cell, organ or tissue levels, but it can also be used to compare proteomes under various conditions of stress, such as those resulted from the exposure to heavy metals [13]. Metabolomics focuses on a global profile of the low molecular weight (<1 000 Da) metabolites which are the end products of metabolisms in biofluids, tissues and even whole organism [14], [15]. The characterization of endogenous metabolites can then provide the information of metabolic status in an organism to assess the biological responses induced by exogenous factors [16], [17]. Both proteomics and metabolomics are frequently used to characterize the perturbations in metabolic pathways and corresponding enzymes and stress-responsive proteins induced by exogenous factors [18]–[20]. Obviously, a combination of proteomics and metabolomics could potentially validate and complement one another, when testing the toxicological effects of environmental pollutants in organisms, especially in non-model organisms [21]. To our knowledge, however, no attempt has been made to evaluate the influence of environmental salinity on the toxicological effects of heavy metal contaminants in the intertidal zones using an integrated proteomic and metabolomic approach at molecular levels.

In this study, two “-omic” approaches, traditional 2-DE-based proteomics and proton nuclear magnetic resonance (NMR) spectroscopy-based metabolomics were used to investigate the chronic effects and corresponding proteomic and metabolic biomarkers induced by environmentally relevant mercury (Hg^2+^, 20 µg L^−1^) in *S. salsa* co-exposed without or with an environmentally relevant salinity (NaCl, 500 mM). In addition, the antioxidant status was assessed in *S. salsa* leaves. Taking all of these outcomes into account, we made an effort to answer three questions. Firstly, what effects can be induced by environmental relevant mercury in *S. salsa* on the basis of metabolic profiles, protein changes and antioxidant enzyme activities? Secondly, what influence can be caused by environmental salinity on the effects of mercury in *S. salsa*? Thirdly, are there any stable proteomic and/or metabolomic biomarkers of mercury-induced effects in *S. salsa* under the experimental salinity?

## Materials and Methods

### Plant Materials and Experimental Conditions

The seeds of *S. salsa* were collected from the wetland located in the Yellow River Delta near the Nature Reserve in November, 2010 and stored in a refrigerator at 4°C. No specific permissions were required for these locations/activities. The location is not privately-owned or protected in any way. The field studies also did not involve endangered or protected species. After surface sterilization in 0.5% HgCl_2_, forty seeds were washed in sterilized double distilled water for three times and sown in the sterilized sands in 4 replicate polyvinylchloride cylinders (20 cm diameter, 20 cm depth). The sown seedlings (*n* = 10) irrigated with Hoagland’s solution (containing 0.1% sodium chloride, pH = 6.0) were divided into four groups: (1) control, (2) Hg^2+^ (20 µg L^−1^), (3) NaCl (500 mM) and (4) combined Hg^2+^ (20 µg L^−1^) and NaCl (500 mM). The experimental concentrations of mercury and salinity were environmentally relevant to the real situation of polluted intertidal zones in the YRD [3], [22]. Mercury concentrations were ranged from 19.3 to 20.3 µg L^−1^ determined by an atomic fluorescence spectrometer (SK-2002A, Jinsuokun LTD., Beijing, China). The seedlings were cultivated at 28±4°C with the photoperiod of 12 h light/12 h darkness, relative humidity 70% and photo-synthetically active radiation 600 µmol m^−2^ s^−1^. After exposure for 30 days, the seedlings (*n* = 6) of *S. salsa* from both control and exposed groups were randomly harvested. After quick measure of fresh weight and total length of seedlings, all plant leaf tissues were collected and flash-frozen in liquid nitrogen and stored at −80°C before extraction for proteins, total RNA and metabolites and enzymatic assay.

### Protein Extraction

Denaturing protein extraction was performed based on the method of Hajheidari et al and Saravanan et al [23], [24]. The frozen leaf tissue was homogenized in liquid nitrogen, resuspended in cold buffer (10% w/v trichloroacetic acid in acetone contained 0.07% w/v DTT). The samples were precipitated overnight at −20°C, centrifuged at 15 000 g for 30 min at 4°C, and the supernatant was discarded. The pellets were resuspended in cold acetone containing 0.07% w/v DTT, then kept at −20°C for 1 h and centrifuged at 15 000 g for 30 min at 4°C. This procedure was repeated twice. The pellets were solubilized in the lysis buffer (7 M urea; 2 M thio urea; 4% m/V CHAPS; 65 mM DTT and 0.2% W/V Bio-lyte buffer) and then incubated for 3 h at room temperature [25]. The homogenate was centrifuged at 15 000 g for 10 min and the supernatant was used for electrophoresis. The total protein was determined by Protein Assay Kit of Tiangen (Beijing, China) using bovine serum albumin as a standard.

### Two-dimensional Gel Electrophoresis

The first dimension (IEF) was performed using the Immobiline Drystrip (24 cm, pH 4–7, linear). One hundred and seventy microgram (170 µg) of proteins was loaded. Isoelectric focusing gel solution containing 7 M urea, 2 M thio urea, 4% m/v CHAPS, 65 mM DTT, 0.001% m/v Bromophenol blue and 0.2% w/v Bio-lyte buffer. IEF was conducted at 20°C with an Etan IPGphor3 system for a total of 90 000 Vh. After the first dimension, strips were placed in the equilibration buffer (0.05 M Tris-HCl, pH 8.8; 6 M urea; 30% glycerol; 2% w/v SDS; containing 1% w/v DTT) and were slowly shaken for 13 min. Subsequently, the strips were incubated for 13 min in the same equilibration buffer with 2.5% (w/v) iodoacetamide without DTT [26]. The second dimension was performed on 10% SDS-PAGE gels using the Ettan DALTsix system (GE Healthcare). After electrophoresis, the gels were silver stained by following the method of Mortz et al and Gharahdaghi et al [27], [28]. Images were captured by ImageScanner III and spots were quantitatively analyzed by using ImageMaster 2D Platinum 7.0. The abundance of each protein spot was estimated by the percentage volume (% vol).

### In Gel Digestion and MS Analysis

In gel digestion was performed according to Katayama et al [29]. Protein spots were washed three times with ultrapure water and de-stained with 25 mmol/L NH_4_HCO_3_ in 50% v/v acetonitrile at room temperature for 30 min. The gels were dried in 50% acetonitrile for 30 min and 100% acetonitrile for another 30 min. The samples were rehydrated in 10 µL cover solution (0.02 g L^−1^ w/v trypsin, 25 mM NH_4_HCO_3_ and 10% acetonitrile) for 30 min, and then covered with the same solution but without trypsin for digestion overnight at 37°C. The supernatants were extracted with 5% TFA in 67% acetonitrile at 37°C for 30 min, then centrifuged at 5 000 g for 5 min, so the peptide extracts and the supernatant of the gel spot were combined.

After being completely dried, the samples were resuspended with 5 µL 0.1% TFA followed by mixing in 1∶1 ratio with a saturated solution of α-cyano-4-hydroxy-trans-cinnamic acid in 50% acetonitrile [30]. One microliter of mixture was analyzed by an ABI 4800 MALDI-TOF/TOF Plus mass spectrometer (Applied Biosystems, Foster City, USA), and data were acquired in a positive MS reflector using a CalMix5 standard to calibrate the instrument (ABI4800 Calibration Mixture). Both the MS and MS/MS data were integrated and processed using the GPS Explorer V3.6 software (Applied Biosystems, USA) with default parameters. Proteins were successfully identified based on 95% or higher confidence interval of their scores in the MASCOT V2.4 search engine (Matrix Science Ltd., London, U.K.). The following parameters were used in the search: NCBInr Viridiplantae (Green Plants) database; trypsin as the digestion enzyme; one missed cleavage site; partial modifications of cysteine carbamidomethylation and methionine oxidization; no fixed modifications; 0.15 Da for precursor ion tolerance and 0.25 Da for fragment ion tolerance. Individual ions scores >40 indicated identity or extensive homology (*P*<0.05).

### Metabolite Extraction

Polar metabolites were extracted from leaf tissues using an extraction system of methanol/water (1/1) as described previously [31]. Briefly, the tissue sample (approximately 300 mg) was ground in a liquid N_2_-cooled mortar and pestle. The tissue powder was thoroughly homogenized in 3.33 mL g^−1^ methanol/water (1/1) using a high throughput homogenizer, Precellys 24 (Bertin, France). After homogenization, the sample was transferred to an Eppendorf tube and centrifuged for 10 minutes (3 000 g, 4°C). The supernatant was removed and then lyophilized. It was subsequently re-dissolved in 600 µL phosphate buffer (0.1 M Na_2_HPO_4_ and NaH_2_PO_4_, containing 0.5 mM TSP, pH 7.0) in D_2_O. The mixture was vortexed and then centrifuged at 3 000 g for 5 min at 4°C. The supernatant substance (550 µL) was pipetted into a 5 mm NMR tube for ^1^H NMR spectroscopic analysis.

### NMR Spectroscopy

Tissue extracts of *S. salsa* were analyzed on a Bruker AV 500 NMR spectrometer at 500.18 MHz (at 298 K) [31]. One dimensional (1D) ^1^H NMR spectra were obtained using a 11.9 µs pulse, 6009.6 Hz spectral width, mixing time 0.1 s, and 3.0 s relaxation delay with standard 1D NOESY pulse sequence, with 128 transients collected into 16 384 data points. Datasets were zero-filled to 32 768 points, and exponential line-broadenings of 0.3 Hz were applied before Fourier transformation. All ^1^H NMR spectra were phased, baseline-corrected, and calibrated (TSP at 0.0 ppm) manually using TopSpin (version 2.1, Bruker). The metabolites were identified and quantified following tabulated chemical shifts and using the software, Chenomx (Evaluation Version, Chenomx Inc., Canada) [32]. The metabolite concentrations were normalized to the mass of leaf tissue by calculating the concentration of metabolites in each NMR tube.

### Data Pre-processing and Multivariate Analysis

One dimensional proton NMR spectra were converted to a format for multivariate analysis using custom-written ProMetab software in Matlab (version 7.0; The MathsWorks, Natick, MA) [33]. Each spectrum was segmented into 0.005 ppm bins between 0.2 and 10.0 ppm with bins from 4.70 to 5.20 ppm (water) excluded from all the NMR spectra. The total spectral area of the remaining bins was normalized to unity that could facilitate the comparison between the spectra. All the NMR spectra were generalized log transformed (glog) with transformation parameter λ = 1.0×10^−9^ [33] to stabilize the variance across the spectral bins and to increase the weightings of the less intense peaks. Data were mean-centered before principal components analysis (PCA).

PCA is an exploratory unsupervised pattern recognition method of analysis which is blind to the status of each sample, and serves to reduce the dimensionality of the data and summarize the similarities and differences between control and treatment groups [8]. The algorithm of this pattern recognition method calculates the highest amount of correlated variation along PC1, with subsequent PCs containing correspondingly smaller amounts of variance. For each model built, the loading vector for the PC was examined to identify the metabolites which contributed to the clusters. For proteomic data, PCA was conducted on the percentage volume (% vol) of common protein spots (matched protein spots in all 2-DE gels) to discover the proteomic differences between different treatments using software PLS Toolbox (version 4.0, Eigenvector Research, Manson, WA). One way analysis of variance (ANOVA) was conducted on the PC scores from each group to test the statistical significance (*P*<0.05) of separations. SAM software was then used to find significant metabolic differences from various groups with appropriate false discovery rate (FDR <0.01) cutoffs [34]. Especially, only those proteins with significant changes (>2.0 folds, *P*<0.05) were considered to be differentially expressed proteins.

### Measurement of Antioxidant Enzyme Activities

The antioxidant enzymes for the activity measurement included superoxide dismutase (SOD, EC 1.15.1.1), peroxidase (POD, EC 1.11.1.7), and catalase (CAT, EC 1.11.1.6). The leaf tissue of *S. salsa* was homogenized in cold buffer (10% w/v phosphate buffer, pH = 7.8), and the antioxidant enzyme activities were assayed by a Multiskan spectrum microplate spectrophotometer (Infinite M200, TECAN) according to the manufacturer’s protocols using the enzyme kits (Jiancheng, Nanjing, China). All the enzyme activities were expressed as units per milligram protein (U/mg protein).

### Total mRNA Extraction and Gene Quantification

The expression of the housekeeping genes (Table 1) in *S. salsa* was determined by qRT-PCR. The data were analyzed with geNorm to calculate the expression stability (M values) of potential reference genes required for accurate normalization (V values) [35]. GeNorm identified β-actin as the most stable gene with a V2/3 value 0.139 less than the proposed geNorm cutoff value of 0.15, which was then followed by 18S ribosomal RNA, helicase, polyubiquitin, ubiquitin, histone. Therefore β-actin was used as the internal control for gene expression normalization.

**Table 1:** The list of primers used for the determination of internal control and quantification of gene expressions by qRT-PCR.

Gene	Forward primer (5′–3′)	Reverse primer (5′–3′)
**Tested genes:**		
heat shock protein 70	GTTCAAGGCAAAAGCACA	GGTGTTTTTGTCTATTCCTTGG
aspartate aminotransferase isozyme 5	TTTCAGTCGGGTCAGCAT	CGAGCATAAGATTCTCTGCCT
S-adenosylmethionine synthase 2	CACCTTAACCCATCTGGC	GACGATGTAGGCACCACT
transketolase	CCTTTGGATGGGAGAAGATCG	GTGACTTAGCTGCTTCGACAAC
peroxiredoxin	TACCCTGCTGATGAAACC	CCCACTGATACCAATAACCT
formate dehydrogenase	ATTTCCCCCCAATTTGTTTCAGAAG	ATTACCCAAAATCAACAAGCCACAC
plastid glutamine synthetase GS2	CTCCATACACCGACAAGT	TGACCCATCATAGTTCCAC
ribulose-1,5-bisphosphate carboxylase/oxygenase	TCTTAACAAGGGATGGGTT	ATCGGATGAATGATTCTGG
chlorophyll A/B binding protein	GCTGATCCGATCCTGAAAG	AAGCAGCGAGCAGGTAGAAC
carbonic anhydrase	GCGCACCATTTGCTGAACAAT	AATCGTAGTAACCTCCCT
ATP synthase delta chain	GAGCGGATTGTGCTGATT	CCTCCATTACCATACCTG
**Reference genes:**		
18S ribosomal RNA	CATCGAGTCTTTGAACGCAAGT	TAGGCAATGCCTTGTATACCAC
helicase	TTTTCGAAGGTGGGTGGAAAGAACG	CAACTTCACTTCTCCTTTACCGCGT
polyubiquitin	CAGACCAGCAGAGGTTGATC	ACGAAGACGAAGCACCAAG
ubiquitin	TAACTGGCAAGACCATCAC	CAAGGTGAAGAGTTGACTCC
histone	CAACAGCGATAAACTACCA	GATTATGGCGTATGGAGA
β-actin	CCGCAAAGATTACATACC	TCACCGAAAGTGCTTCTA

Total RNA from leaf tissues of *S. salsa* was isolated using the Trizol reagent (Invitrogen). Single-strand cDNA was synthesized from total RNA with M-MLV reverse transcriptase (Promega, USA). Gene-specific primers for heat shock protein 70, aspartate aminotransferase, S-adenosylmethionine synthase, transketolase, peroxiredoxin, formate dehydrogenase, glutamine synthetase GS2, ribulose-1,5-bisphosphate carboxylase/oxygenase (rubisco), chlorophyll A/B binding protein, carbonic anhydrase and ATP synthase delta chain were used to amplify amplicons specific for *S. salsa* samples. The sequences of primers are given in Table 1.

The quantitative real time RT-PCR amplifications were carried out in triplicate with a total volume of 50 µL containing 25 µL of 2×SYBR Green PCR Master Mix, 20 µL of the diluted cDNA, 1 µL of each of primers (10 µmol/L), and 3 µL of DEPC-treated water. The quantitative real time RT-PCR program was as follows: 50°C for 2 min, 95°C for 10 min followed by 40 cycles of 95°C for 15 s and 60°C for 1 min. After PCR program, the data were analyzed with the ABI 7500 SDS software (Applied Biosystems). The comparative CT method (2^−ΔΔCT^) was used to analyze the expression levels of genes [36].

### Statistical Analysis

Data of growth parameters, enzyme activities, gene expressions and metabolite concentrations were expressed as the mean ± standard deviation (*n* = 6). The Minitab software (Version 15, Minitab Inc., USA) was used for the statistical analysis. One way analysis of variance (ANOVA) with Tukey’s test was conducted on the data, and a *P* value at 0.05 was considered significant.

## Results

### The Growth Data of *S. salsa* from Control and Treatment Groups

**Figure 1:**
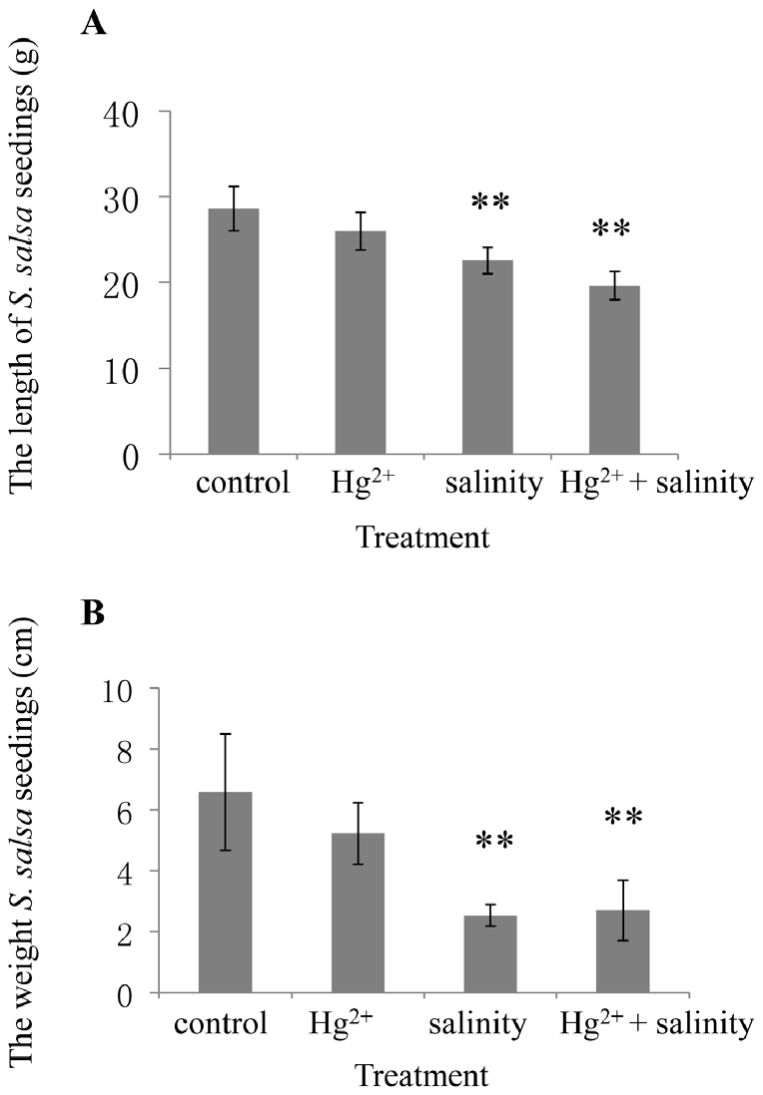
The average length (A) and fresh weight (B) of *S.* *salsa* seedlings from control, Hg^2+^−, salinity- and Hg^2+^+salinity-treated groups.

The average lengths and fresh weights of *S. salsa* seedlings were shown in Figure 1. Obviously Hg^2+^ exposure (20 µg L^−1^) did not significantly (*P*>0.05) affect the growth of *S. salsa*. However, both salinity and Hg^2+^+salinity treatments significantly inhibited the growth of *S. salsa* after exposure for 1 month.

### Proteomic Responses in Leaf Tissues of *S. salsa*

**Figure 2:**
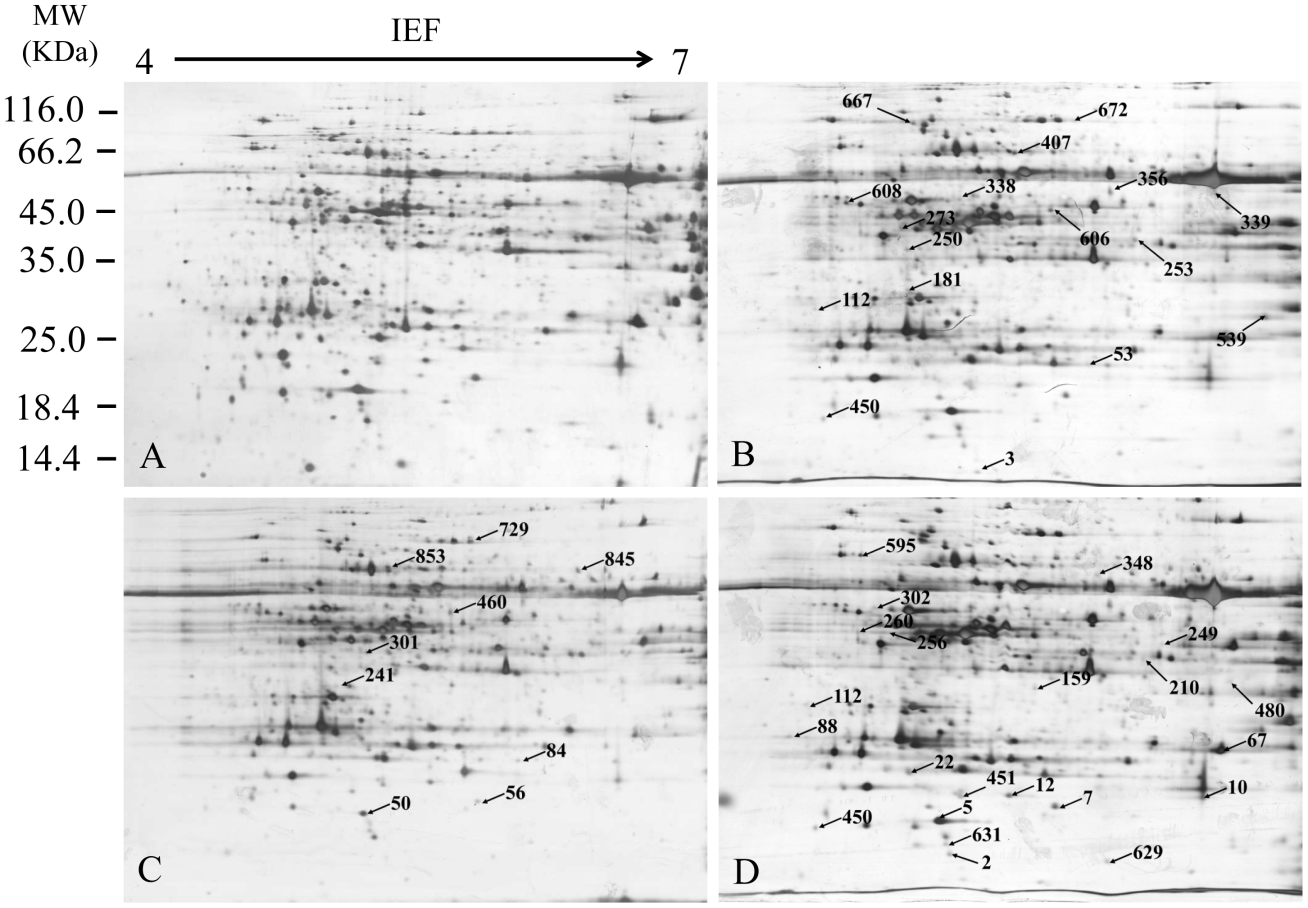
Representative 2-DE images of proteins extracted from *S. salsa* leaves. Proteins were submitted to isoelectric focusing on 4–7 IPG strips (24 cm) followed by electrophoresis on 10% SDS-PAGE. Gels were stained by silver stain. Gels are from the exposed groups of control (A), Hg^2+^ (20 µg L^−1^) (B), salinity (NaCl, 500 mM) (C) and Hg^2+^+salinity (D).

Comparative proteomic analysis was used to determine the proteomic profiles in *S. salsa* exposed to Hg^2+^, salinity and combined Hg^2+^ and salinity (designated as Hg^2+^+salinity). A total of 60 spots resolved in 2-DE gels were differentially expressed (>2 folds, *P*<0.05). Figure 2 represented the differential protein spots in *S. salsa* with different exposures. In the present study, the proteins spots observed in all three biological replicates of silver-stained gels were analyzed by MALDI-TOF/TOF mass spectrometry and 43 (∼70%) proteins were successfully identified. The details of matched proteins including their protein names, accession numbers and other MS data are summarized in Table 2.

**Table 2:** List of protein spots which were differentially expressed in *S. salsa* leaves under mercury, salinity treatment after 30 days.

spot ID.^d^	protein name	plant species	gi number^e^	MW/kDa^f^	PI	score^g^	SC^h^	PN^i^	fold changes^k^
**metabolism**									
407	2, 3-bisphosphoglycerate-independent phosphoglycerate mutase	*Solanum tuberosum*	gi|548533	61257	5.98	91	2%	1	−2.07191^ a^
539	glyceraldehyde 3-phosphate dehydrogenase	*Ricinus communis*	gi|255539282	49132	7.56	485	15%	5	−10.1754^ a^
253	aspartate aminotransferase isozyme 5	*Glycine max*	gi|7548843	50698	7.71	351	12%	4	−3.88862^ a^
273	succinyl CoA ligase beta subunit-like protein	*Solanum tuberosum*	gi|83284007	45582	6.63	144	6%	3	−2.59542^ a^
608	S-adenosylmethionine synthase 2	*Solanum lycopersicum*	gi|127046	43618	5.57	391	16%	5	−2.69496^ a^
339	beta-ketoacyl-ACP synthase I-1	*Arachis hypogaea*	gi|210110274	50467	8.46	247	13%	3	−2.86286^ a^
672	transketolase	*Platanus x acerifolia*	gi|110224784	25954	6.25	159	11%	2	−4.44503^ a^
									−5.1985^ b^
241	spermidine synthase	*Panax ginseng*	gi|251831262	36765	5.02	262	11%	3	2.08093^ b^
301	cysteine synthase	*Spinacia oleracea*	gi|303902	40896	6.79	193	8%	2	2.17962^ b^
460	1-deoxy-D-xylulose 5-phosphate reductoisomerase	*Stevia rebaudiana*	gi|22797429	51502	5.97	223	8%	3	−2.04051^ b^
302	fructose-1, 6-bisphosphatase	*Arabidopsis thaliana*	gi|297816710	45606	5.41	610	19%	5	10.7831^ c^
159	putative glyoxalase	*Oryza sativa Japonica Group*	gi|46485858	29720	4.99	104	6%	1	2.50926^ c^
260	pantothenate kinase, putative	*Ricinus communis*	gi|255546706	41379	4.92	289	13%	4	3.90264^ c^
249	sulfate adenylyltransferase	*Ricinus communis*	gi|255538896	47839	5.94	77	5%	2	2.71891^ c^
348	adenosylhomocysteinase	*Mesembryanthemum crystallinum*	gi|6094228	53771	5.75	157	6%	2	4.20273^ c^
256	plastid glutamine synthetase GS2	*Solanum tuberosum*	gi|209529860	47902	6.68	349	14%	1	2.74086^ c^
480	formate dehydrogenase	*Ricinus communis*	gi|255552590	42868	6.28	112	6%	2	−7.79127^ c^
**protein biosynthesis**									
450	50S ribosomal protein L12	*Spinacia oleracea*	gi|133085	19922	5.5	115	7%	1	−3.92588 ^a^
									−4.83338 ^c^
356	elongation factor 1-gamma 3	*Zea mays*	gi|195628630	47357	6.01	146	6%	2	2.78465^ a^
250	elongation factor Tu	*Vitis vinifera*	gi|225456880	52830	6.25	357	10%	3	−4.98755^ a^
338	eukaryotic initiation factor 4A-11	*Vitis vinifera*	gi|303844	47119	5.46	340	16%	4	4.06876^ a^
112	elongation factor 1 beta’	Oryza sativa Japonica Group	gi|218161	23815	4.86	90	5%	1	−2.62973^c^
**stress and defense**									
53	dehydroascorbate reductase-like protein	*Solanum tuberosum*	gi|76160951	23596	6.09	219	13%	2	5.02688^ a^
									9.64959^ b^
606	salt tolerance protein 2	*Beta vulgaris*	gi|39725278	38430	5.36	107	5%	1	−2.13402^ a^
667	heat shock protein 70	*Gossypium hirsutum*	gi|224112795	71486	5.1	363	8%	4	−2.01^ a^
2	peroxiredoxin, putative	*Salsola tragus*	gi|255575353	29551	5.56	417	20%	3	−12.6443^c^
**signaling pathway**									
136	14-3-3 protein	*Manihot esculenta*	gi|291293221	29902	4.79	346	26%	5	−4.28204^a^
10	auxin-binding protein ABP19a	*Vitis vinifera*	gi|225444754	22929	6.88	158	10%	2	−8.99942^ c^
**energy**									
3	ATP synthase CF1 epsilon subunit	*Calycanthus floridus var. glaucus*	gi|32480849	14515	5.84	115	8%	2	−34.4084^ a^
853	ATP synthase CF1 alpha subunit	*Nicotiana tomentosiformis*	gi|81301547	55401	5.14	563	16%	5	−4.89946^ b^
845	ATPase subunit	*Beta vulgaris subsp. vulgaris*	gi|11263	55306	5.69	202	5%	2	−2.93973^ b^
451	ATP synthase delta chain	*Populus trichocarpa*	gi|118485281	11550	4.94	182	25%	5	−2.86363^c^
**photosynthesis**									
5	magnesium-chelatase subunit chII,chloroplast precursor, putative	*Ricinus communis*	gi|255563060	21747	7.86	255	8%	2	−2.61262^ c^
									−5.658^ b^
7	ribulose-1, 5-bisphosphate carboxylase/oxygenase large subunit	*Chenopodium sanctae-clarae*	gi|34576565	50120	6.13	488	22%	6	−6.57158^ c^
									−4.73659^ b^
629	ribulose bisphosphate carboxylase	*Oryza sativa*	gi|20341	19741	8.26	66	4%	1	−8.69256^ c^
12	chlorophyll A/B binding protein, putative	*Medicago truncatula*	gi|217071344	31249	6.59	297	12%	4	−3.35594^ c^
67	carbonic anhydrase	*Spinacia oleracea*	gi|115472	34947	6.61	288	14%	4	−3.61204^ c^
**protein folding and assembly**									
88	chloroplast RNA binding protein	*Mesembryanthemum crystallinum*	gi|168274276	33785	4.68	518	30%	7	−11.2616^c^
595	putative protein disulfide isomerase	*Gossypium raimondii*	gi|133902301	55845	4.92	68	1%	1	−2.34923^c^
**immunosuppression**									
631	putative cyclophilin	*Oryza sativa Japonica Group*	gi|53792605	22268	9.12	51	6%	1	−4.07635^ c^
**unkown**									
181	Os04g0490800	*Oryza sativa Japonica Group*	gi|115459134	39811	6.75	150	10%	4	2.23461^ a^
210	hypothetical protein SELMODRAFT_233332	*Selaginella moellendorffii*	gi|302795279	35712	8.12	57	3%	1	2.94349^ c^
22	hypothetical protein VITISV_002160	*Vitis vinifera*	gi|147772909	34502	6.15	81	5%	1	−2.45227^ c^

a, b, c Identification of differentially expressed proteins compared with control and Hg^2+^ (20 µg L^−1^), salinity (NaCl, 500 mM) and Hg^2+^+salinity, respectively after 28 days. ^d^ Assigned spot ID as indicated in **Figure 1**. ^e^ Database accession numbers after searching against the NCBInr database. ^f^ Experimental mass. ^g^ Mascot score reported. ^h^ Sequence coverage. ^i^ Number of peptide sequences. ^k^ Fold changes with significant changes (>2-fold, *P*<0.05 ) were calculated using ImageMaster 2D Platinum 7.0.

**Figure 3:**
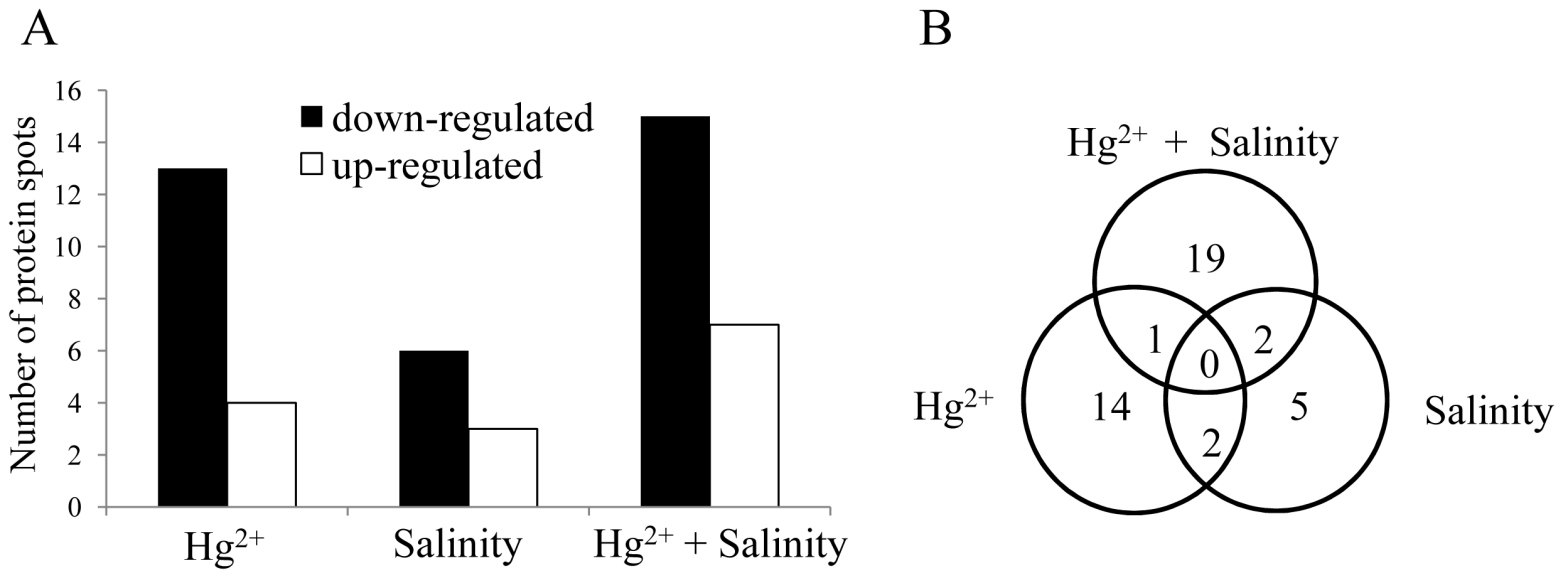
Number of protein spots from *S.* *salsa* samples (A) and the differentially expressed proteins in *S. salsa* from Hg^2+^ (20 µg L^−1^), salinity (NaCl, 500 mM) and Hg^2+^+salinity (B).

In Hg^2+^-treated group, a total of 17 differentially expressed spots were discovered, including 4 up-regulated and 13 down-regulated. These proteins were related to metabolism, protein biosynthesis, stress and defense, signaling pathway and energy metabolism (Table 2). Nine differentially expressed proteins were observed in salinity-treated *S. salsa* samples, including 3 up-regulated and 6 down-regulated proteins that were involved in metabolism, photosynthesis, energy metabolism and stress and defense. For the Hg^2+^+salinity-treated group, a total of 22 proteins (7 up-regulated and 15 down-regulated) were significantly altered. All these differentially expressed proteins from different treatments were basically involved in metabolism, photosynthesis, protein folding and assembly, protein biosynthesis, stress and defense, signaling pathway, immunosuppression and energy metabolism. Figure 3 revealed the total numbers of protein spots differentially expressed from *S. salsa* samples with various treatments.

### The Relationship between Gene Expressions and Protein Abundances

**Figure 4:**
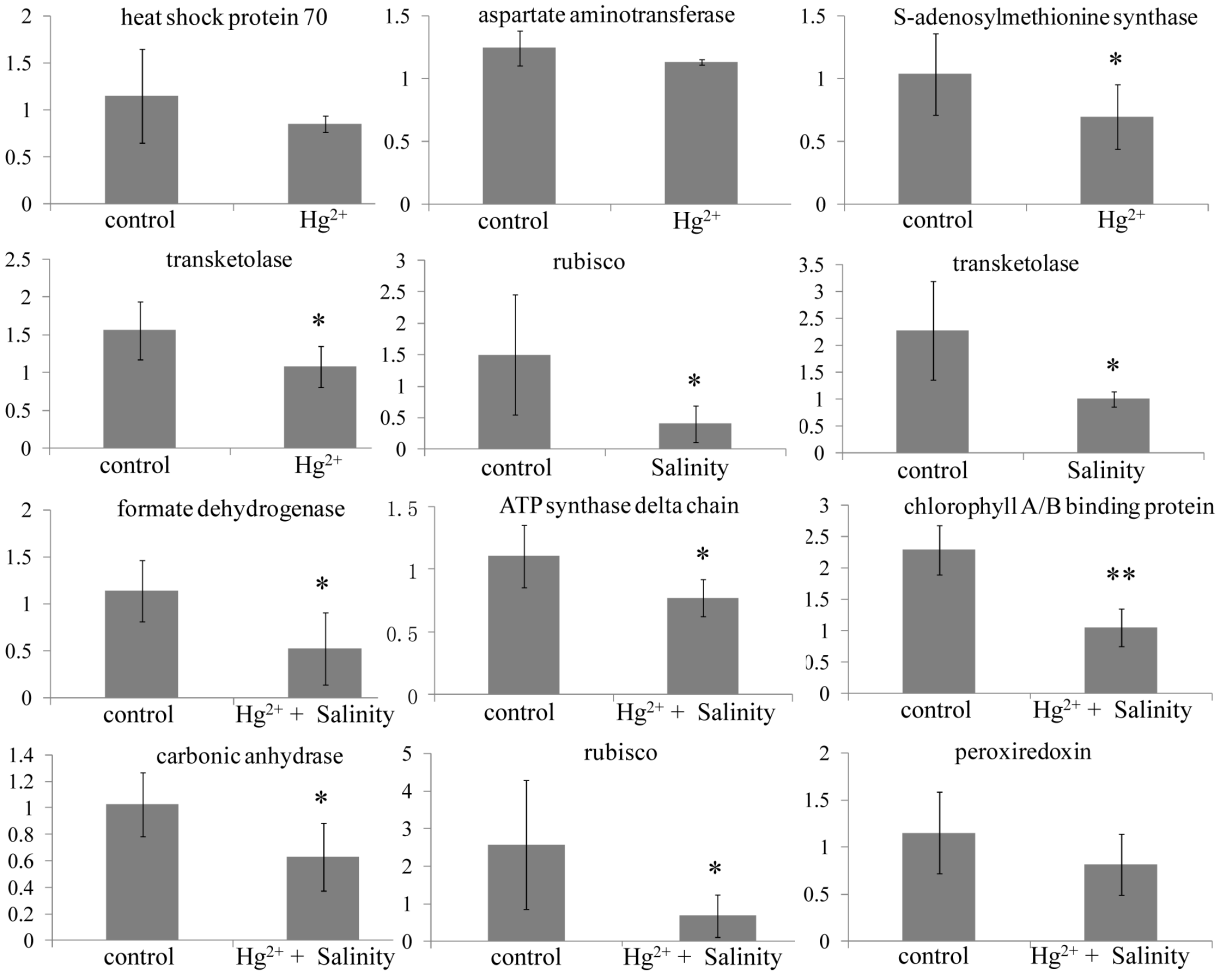
Expression levels of eleven genes in leaf tissues of *Suaeda salsa* from control, Hg^2+^ (20 µg L^−1^), salinity (NaCl, 500 mM) and Hg^2+^+salinity-treated groups after exposure for 30 days. Data (*n* = 6) were expressed as mean ± standard deviation. Significant difference between control and exposed groups was tested by one-way analysis of variance with Tukey’s test and indicated by *(*P*<0.05) and **(*P*<0.01).

To further verify the results of 2-DE and compare the correlation between gene expression and protein abundances, eleven representative genes related to regulated proteins were quantified using quantitative real-time RT-PCR technique. The results indicated that the expression levels of the genes had similar alteration tendency with corresponding proteins (Figure 4).

### Metabolic Responses in Leaf Tissues of *S. salsa*

**Figure 5:**
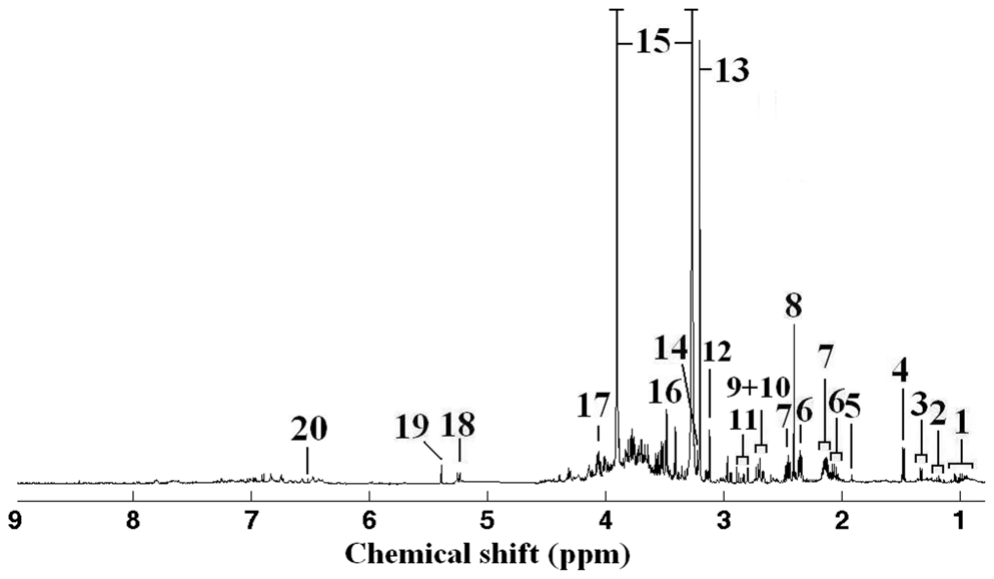
One representative 1-dimensional 500 MHz ^1^H NMR spectrum of metabolite extracts of the leaf tissue from *S.* *salsa* in control group using extraction solvent system of methanol/water (1/1). Metabolite assignments: (1) branched chain amino acids: leucine, isoleucine and valine, (2) ethanol, (3) threonine, (4) alanine, (5) acetate, (6) glutamate, (7) glutamine, (8) succinate, (9) malate, (10) aspartate, (11) asparagine, (12) malonate, (13) choline, (14) phosphocholine, (15) betaine, (16) glycine, (17) fructose, (18) glucose, (19) unknown (5.39 ppm) and (20) fumarate.

Figure 5 shows one representative ^1^H NMR spectrum of leaf tissue extracts from the control group. Different classes of metabolites were identified in leaf tissue of *S. salsa*, including amino acids (branched chain amino acids: valine, leucine and isoleucine, alanine, threonine, glutamate, aspartate, asparagine, glycine, etc.), sugars (fructose and glucose), an organic osmolyte (betaine), organic acids (acetate and malonate) and intermediates in the tricarboxylic acid (TCA) cycle (succinate, malate and fumarate). Especially, all NMR spectra were dominated by the osmolyte, betaine that is synthesized from choline by choline monooxygenase [37]. In the higher plant, halophyte *S. salsa*, betaine is the most important secondary metabolite playing a pivotal role in the protection from osmotic stress [38].

PCA resulted in significant separation (*P*<0.05) control and each treatment (data not shown), which indicated that Hg^2+^, salinity and Hg^2+^+salinity induced significant effects in *S. salsa*. Especially, the inhibited plant growth data confirmed the effects by salinity and Hg^2+^+salinity exposures (Figure 1). For further analysis, one way ANOVA was directly conducted on the metabolite concentrations to explore the significant metabolic responses induced by these treatments. For Hg^2+^-treated *S. salsa* group, the metabolic profiles of leaf tissue extracts indicated significant (*P*<0.05) increases of branched chain amino acids (valine, leucine and isoleucine) and phosphocholine, and significant (*P*<0.05) decreases of ethanol, acetate, succinate, malonate and choline (Table 3). The significant metabolic differences induced by salinity in the leaf tissues of *S. salsa* included the depleted threonine, alanine and acetate, and elevated malate, choline, betaine, fructose and glucose (Table 3). For Hg^2+^+salinity-treated *S. salsa* samples, the significant metabolic changes resulted in decreases of ethanol, acetate, succinate, malonate and glycine, and increases of valine, asparagine, phosphocholine and betaine.

**Table 3:** Metabolite concentrations (µmol g^−1^ wet tissue) in leaf tissues of *S. salsa* exposed to Hg^2+^ (20 µg L^−1^), salinity (NaCl, 500 mM) and combined Hg^2+^ and salinity.

Metabolites^a^	Chemical shift(ppm, multiplicity)	Exposures			
		Control	Hg^2+^	Salinity	Hg^2+^+Salinity
Valine	1.05 (d)	0.052±0.008	0.145±0.019**	0.055±0.004	0.070±0.006**
Isoleucine	1.00 (d)	0.042±0.007	0.130±0.019**	0.036±0.008	0.051±0.011
Leucine	0.94 (t)	0.035±0.007	0.102±0.018**	0.034±0.007	0.035±0.008
Ethanol	1.19 (t)	0.044±0.017	0.022±0.003*	0.030±0.007	0.024±0.006*
Threonine	1.34 (d)	0.091±0.013	0.121±0.053	0.078±0.007*	0.081±0.013
Alanine	1.48 (d)	0.310±0.090	0.408±0.088	0.186±0.027**	0.298±0.063
Acetate	1.91 (s)	0.018±0.002	0.012±0.003**	0.013±0.002**	0.011±0.002**
Glutamate	2.05 (m)	1.098±0.152	1.268±0.200	1.514±0.341	1.460±0.162
Glutamine	2.14 (m)	0.690±0.536	0.703±0.214	0.777±0.167	0.864±0.268
Malate	2.68 (dd)	0.522±0.229	0.508±0.365	1.404±0.738*	0.711±0.211
Succinate	2.41 (s)	0.440±0.117	0.184±0.050**	0.495±0.201	0.229±0.076**
Aspartate	2.68 (ABX)	0.493±0.090	0.437±0.098	0.562±0.107	0.463±0.110
Asparagine	2.85 (ABX)	0.277±0.092	0.346±0.108	0.431±0.152	0.536±0.137**
Malonate	3.13 (s)	0.075±0.012	0.061±0.008*	0.066±0.012	0.048±0.019*
Choline	3.19 (s)	0.789±0.07	0.660±0.036**	1.062±0.211*	0.768±0.260
Phosphocholine	3.21 (s)	0.050±0.010	0.095±0.023**	0.045±0.025	0.084±0.025*
Betaine	3.27 (s)	17.57±1.69	16.47±1.37	46.81±1.34**	39.59±9.63**
Glycine	3.57 (s)	0.122±0.031	0.091±0.024	0.167±0.038	0.059±0.015**
Fructose	4.01 (m)	0.772±0.07	0.442±0.211	1.71±0.457**	0.452±0.259
Glucose	4.64 (d), 5.23 (d)	2.04±0.707	1.41±0.768	5.02±2.76*	1.28±0.738
Fumarate	6.52 (s)	0.035±0.023	0.021±0.008	0.038±0.008	0.030±0.015

Values are presented as mean ± standard deviation (*n* = 6).

a Statistical significances (*P*<0.05,^*^and *P*<0.01,^**^) between control and heavy metal-exposed *S. salsa* samples were determined by one-way ANOVA.

b s = singlet, d = doublet, dd = double doublet, t = triplet, m = multiplet, ABX = complex multiplet involving 2 protons (A and B) and a heavy atom (X).

### Antioxidant Enzyme Activities

**Figure 6:**
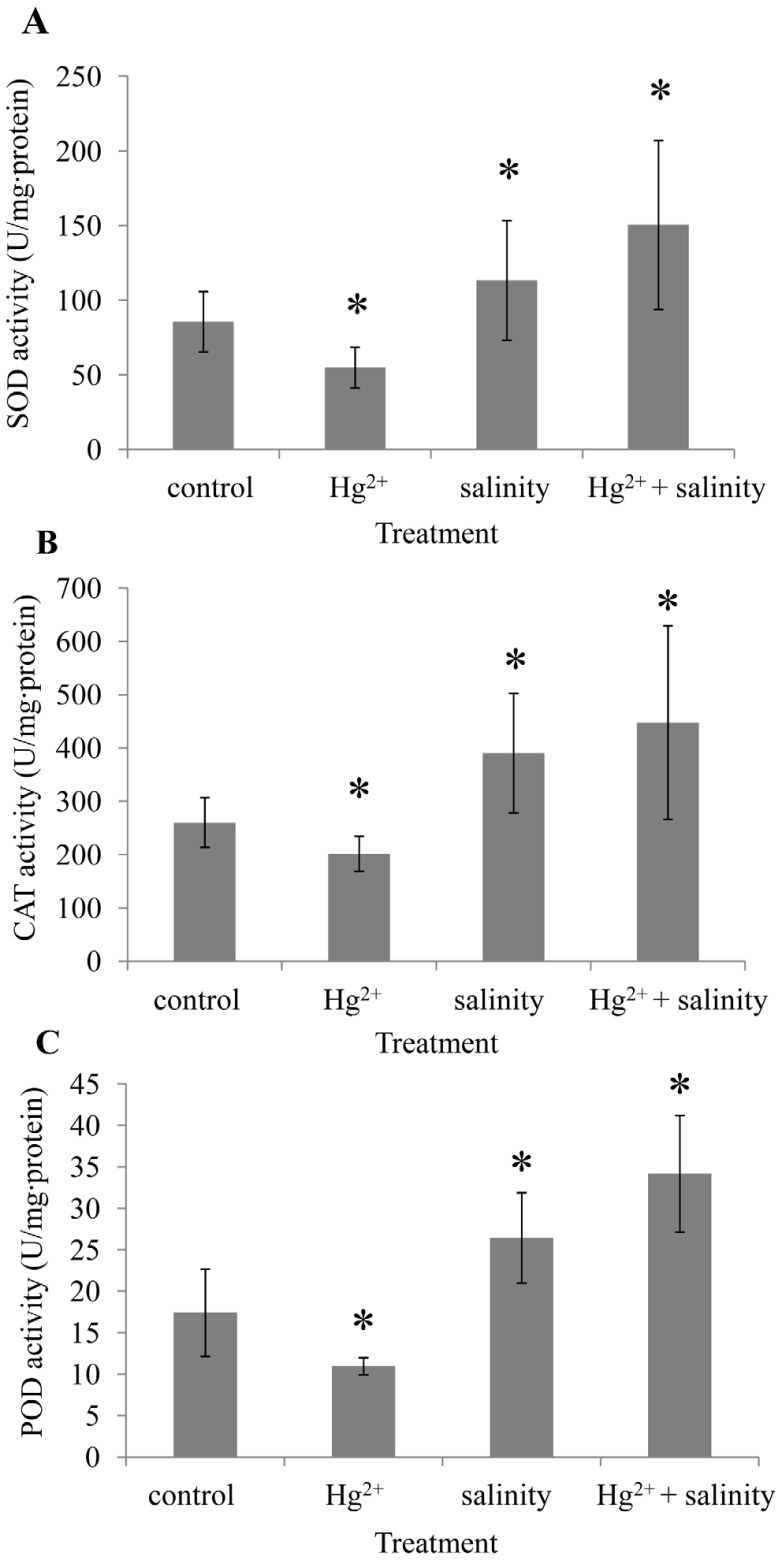
The activities (U mg^−1^ protein) of SOD (A), POD (B) and CAT (C) in leaf tissues from control, Hg^2+^ (20 µg L^−1^), salinity (NaCl, 500) and Hg^2+^+salinity-exposed *S.* *salsa* samples (*n* = 6). Data were expressed as mean ± standard deviation. Significant difference between control and exposed groups was tested by one-way analysis of variance with Tukey’s test and indicated by *(*P*<0.05) and **(*P*<0.01).

After exposure for 30 days, the average activities of antioxidant enzymes including SOD, POD and CAT were significantly (*P*<0.05) increased in both salinity and Hg^2+^+salinity-exposed samples of *S. salsa* (Figure 6). However, Hg^2+^ treatment induced significant (*P*<0.05) decreases in antioxidant enzyme activities in the *S. salsa* samples.

## Discussion

### Effects of Hg^2+^, Salinity and Hg^2+^+Salinity on the Proteome of *S. salsa*

**Figure 7:**
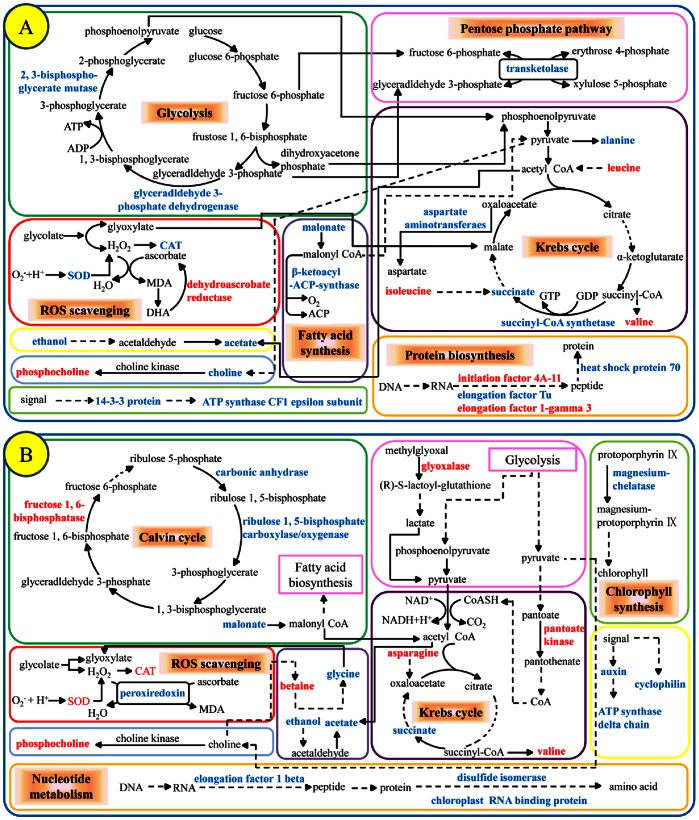
Schematic presentation of molecular responsive-mechanisms in *S.* *salsa* to Hg^2+^ co-exposed without (A) or with (B) the environmentally relevant salinity (NaCl, 500 mM) according to Kyoto Encyclopedia of Genes and Genomes (http://www.genome.jp/kegg/) and Uniprot (http://www.uniprot.org/). The identified proteins and metabolites were shown by marking the protein names in red (up-regulated) or blue (down-regulated).

Figure 7A summarized the pathways involved in the response of *S. salsa* to Hg^2+^. Some proteins related to glycolysis, pentose phosphate pathway, fatty acid synthesis, TCA cycle were changed (decreased or increased) in Hg^2+^-treated *S. salsa* group (Table 2), which clearly demonstrated the disturbance in metabolism caused by Hg^2+^ exposure in *S. salsa* [39]–[45]. One 14-3-3 protein was decreased (>4 folds) in mercury-treated group. 14-3-3 proteins in plant cells participate in signal transduction, regulate the activity of enzymes, and are associated with the ATP synthases, representing a mechanism for plant adaptation to environmental changes [46]–[49]. Especially, ATP synthase CF1 epsilon subunit was dramatically decreased (>34 folds) in Hg^2+^-treated *S. salsa* group. Bunney et al. (2001) reported that 14-3-3 protein is a regulator of the mitochondrial and chloroplast ATP synthase (Figure 7A) [46], therefore, the consistent down-regulation of 14-3-3 protein and ATP synthase CF1 epsilon subunit confirmed the disturbance in energy metabolism caused by Hg^2+^ exposure in *S. salsa*. Salt tolerance proteins can increase salt tolerance in some plants and can be over expressed when plants are exposed to salinities [49]. Interestingly, one salt tolerance protein was significantly down-regulated in Hg^2+^-treated *S. salsa* samples, which might suggest a contrary effect of Hg^2+^ to salinity in *S. salsa*. Heat shock protein 70 (Hsp 70) has multiple functions in preventing aggregation, assisting refolding, protein import and translocation, signal transduction, anti-oxidative stress and transcriptional activation under different environmental stress conditions such as heat, cold and drought [50]. Mercury can induce oxidative stress in organisms. Therefore, *S. salsa* may use various protective antioxidants to resist oxidative stress and probably up-regulates the contents of antioxidants, such as heat shock proteins and anti-oxidative enzyme activities, such as SOD and CAT [51]. Elbaz et al. (2010) reported that mercury (>200 µg L^−1^) could induce significant oxidative stress in *Chlamydomonas reinhardtii* [51]. However, Hsp 70 was down-regulated under Hg^2+^ exposure, together with decreased SOD, CAT and POD activities which were significantly increased in *S. salsa*. Clearly, the environmentally relevant concentration (20 µg L^−1^) of Hg^2+^ induced a contrary result in *S. salsa* compared with that in *Chlamydomonas reinhardtii* Hg^2+^ (>200 µg L^−1^), which probably implied that this relatively low concentration (20 µg L^−1^) of Hg^2+^ induced a hormesis phenomenon of antioxidants (Hsp 70, SOD, CAT and POD) in *S. salsa*. In both salinity- and Hg^2+^+salinity-treated groups, these antioxidant enzymes were significantly increased which apparently indicated the oxidative stress induced by salinity in *S. salsa* but Hg^2+^.

In response to salinity, the identified proteins relating to metabolism were involved in pentose phosphate pathway (transketolase), polyamine biosynthesis (spermidine synthase), amino-acid biosynthesis (cysteine synthase) and isoprenoid biosynthetic process (1-deoxy-D-xylulose 5-phosphate reductoisomerase). The down-regulated ATP synthase CF1 alpha subunit and ATPase subunit implied the disturbance in energy metabolism caused by salinity exposure in *S. salsa* [52]. The similar result of down-regulated ATP synthase was also observed in another halophyte *Thellungiella* exposed to salinities, which could imply the similar responsive mechanism of energy metabolism halophytes to salinity exposures [52].

Figure 7B summarized the proteomic and metabolomic responses involved in the pathways, including Calvin cycle, ROS scavenging, TCA cycle, chlorophyll synthesis, nucleotide metabolism, immunosuppression, signaling pathway and energy metabolism, in *S. salsa* to Hg^2+^+salinity. The proteins associated with metabolism were involved in Calvin cycle, pyruvate metabolism, sulfur metabolism, cysteine and methionine metabolism, methane metabolism, glutamate metabolism, pantothenate and CoA biosynthesi [53]–[57], as shown in Table 2 and Figure 7B. Auxins play a vital role in division and expansion of plant cells, and its action is mediated by the putative receptor called auxin-binding protein [58] which was dramatically down-regulated (∼9 folds) in Hg^2+^+salinity-treated samples. Sauer et al. (2006) found that auxin-binding protein was significantly up-regulated in maize during shoot-borne root initiation, which confirmed that auxin-binding protein was important in plant growth [59]. Therefore, the down-regulation of auxin-binding protein indicated that the co-exposure of Hg^2+^ and salinity inhibited plant growth in *S. salsa*, which was indicated by the reduced (*P*<0.001) plant weight and length of Hg^2+^+salinity-treated samples (Figure 1). Of particular interest is that cyclophilin was found to be decreased under Hg^2+^+salinity stress. Cyclophilin belongs to a family of immunosuppressant receptor proteins presented in all subcellular compartments which are involved in a wide variety of processes such as protein trafficking and maturation and receptor complex stabilization [60]. The down-regulation of cyclophilin probably indicated that multiple cellular pathways were affected by Hg^2+^+salinity exposure. Peroxiredoxin, an antioxidant enzyme reducing hydrogen peroxide (H_2_O_2_) and alkyl hydroperoxides treatments, regulates peroxide-mediated signaling cascades [61], [62]. In another native halophyte *Thellungiella* in the YRD, peroxiredoxin was down-regulated the seedlings of *Thellungiella* was exposed to NaCl (150 mM) [52]. In our case, the single exposure of Hg^2+^ or salinity did not induce obvious alteration of peroxiredoxin in *S. salsa*, however, the combined exposure of Hg^2+^ and salinity significantly down-regulated peroxiredoxin. It might be the synergistic effects between salinity and Hg^2+^ in *S. salsa*. As to protein folding and assembly, chloroplast RNA binding protein and protein disulfide isomerase (PDI) were down-regulated. PDI is a multifunctional enzyme catalyzes a set of disulfide-exchange reaction that includes formation, reduction or isomerization of protein disulfide bonds [63], [64]. In addition, as a member of the superfamily of thioredoxin, PDI has been found in response to oxidative stress [65]. Proteins are the main nonwater components of a cell and they are the major targets attacked by intracellular reactive oxygen species (ROS). As a member of thioredoxin family, PDI can reverse these oxidative modifications. Therefore, the down-regulation of PDI could imply that the Hg^2+^+salinity affected the antioxidant system in *S. salsa*, which is evidenced by the increased activities of CAT, SOD and POD in this treatment.

50S ribosomal protein L12 was down-regulated in both Hg^2+^- and Hg^2+^+salinity-treated groups. The synthesis of this protein occurs in large macromolecular particles called ribosomes [66]. And it is presumed to be involved in the binding of translation factors, stimulating factor-dependent GTP hydrolysis [67]. Transketolase was down-regulated and dehydroascorbate reductase-like protein was up-regulated in both Hg^2+^- and salinity-treated group. Dehydroascorbate reductase (DHAR) plays an important role in maintaining the normal level of ascorbic acid by recycling oxidized ascorbic acid [68], [69]. The increasing dehydroascorbate reductase-like protein is critical for protection of cellular components against mercury and salinity stresses. Proteins linked to the function of photosynthesis (magnesium-chelatase and ribulose-l,5-bisphosphate carboxylase/oxygenase) were decreased in both salinity- and Hg^2+^+salinity-treated groups. Magnesium-chelatase catalyzes the insertion of Mg^2+^ into protoporphyrin IX, the first dedicated step in chlorophyll biosynthesis [70], [71]. Ribulose-l,5-bisphosphate carboxylase/oxygenase acts as a carboxylase with the substrates RuBP and CO_2_ and as an oxygenase with the substrates RuBP and O_2_. The degradation of Rubisco can be a key factor that determined the photosynthetic activity in plants [72], [73]. The down-regulation of magnesium-chelatase and ribulose-l,5-bisphosphate carboxylase/oxygenase demonstrated that Hg^2+^ and salinity stresses could weaken the photosynthesis system of *S. salsa*. Interestingly, the level of magnesium-chelatase in salinity-treated group was significantly lower than that in Hg^2+^+salinity-treated group, which suggested the antagonistic effect between salinity and Hg^2+^ in *S. salsa*. Compared to Hg^2+^+salinity-treated group, however, the relatively high level of ribulose-l,5-bisphosphate carboxylase/oxygenase in salinity-treated group implied the additive effects of Hg^2+^ to salinity.

Essentially, gene expression means the tendency of the corresponding encoded protein that is the down-stream product of the gene. In some cases, however, gene expressions are not always correlated with the protein abundances. The lack of correlation between gene and protein expressions is not strange since there could be different regulatory mechanisms of gene expressions and protein expressions to contaminant exposures. In addition, the protein translation of mRNA transcription does not always happen due to the posttranscriptional modifications. In our case, the expression levels of selected genes were consistent with alteration tendency of corresponding proteins, which could partly confirm the regulations of proteins in *S. salsa* exposed to Hg^2+^, salinity and Hg^2+^+salinity.

### Effects of Hg^2+^, Salinity and Hg^2+^+Salinity on the Metabolome of *S. salsa*

For the leaf tissues of *S. salsa* seedlings exposed to Hg^2+^, the levels of branched chain amino acids (valine, isoleucine and leucine) were approximately 3 times higher than those in the samples of control group (Table 3), which was probably associated with enhanced protein bio-degradation or inhibited protein biosynthesis caused by Hg treatment in *S. salsa* [19]. Ethanol is the end product of anaerobic metabolism through fermentation pathway. Subsequently, ethanol can be catalyzed into acetate by alcohol dehydrogenase and aldehyde dehydrogenase [74]. In this study, both ethanol and acetate were significantly (*P*<0.05) decreased in Hg^2+^-exposed *S. salsa* samples, which indicated that Hg^2+^ exposure inhibited anaerobic metabolism in the leaf of *S. salsa*. Succinate, one of the intermediates involved in tricarboxylic acid (TCA) cycle, was significantly (*P*<0.01) depleted in *S. salsa* samples, indicating the disturbance in TCA cycle that is related to energy metabolism [19]. Interestingly, succinyl-CoA-synthetase that is involved in TCA cycle, was down-regulated as well in Hg^2+^-exposed *S. salsa* samples. Succinyl-CoA-synthetase is an enzyme that catalyzes the reversible reaction of succinyl-CoA to succinate (as shown in Figure 7A). Therefore the consistent alteration of both succinyl-CoA and succinate confirmed that proteomics and metabolomics might validate one another in special metabolic pathways. Choline and ATP can be converted into phosphocholine and ADP catalyzed by choline kinase. Therefore the elevated phosphocholine and reduced choline might indicate the enhanced conversion of choline to phosphocholine and hence enhanced energy cost (ATP→ADP) to prevent *S. salsa* from Hg^2+^-induced damage.

Salinity is a common environmental stressor to plants. Although *S. salsa* is a halophyte that can tolerate high salinities, the extremely high concentration (∼500 mM) of environmental salinity can lead to significant inhibition of plant growth, which was observed in this study (data not shown). At metabolite level, the experimental concentration of salinity (500 mM, also realistically relevant) induced differential metabolic responses in leaf tissues of *S. salsa* compared with those induced by Hg^2+^ exposure. The decreased threonine and alanine indicated possible inhibition of protein bio-degradation or enhanced protein biosynthesis caused by salinity stress in *S. salsa*. The significant decrease of acetate could be related to the inhibited anaerobic metabolism combined with the reduced level of ethanol (although not significant, *P* = 0.11). The intermediate in TCA cycle, malate, was significantly (*P*<0.05) increased, which suggested the disturbance in energy metabolism. Both choline and betaine were significantly increased in salinity-stressed *S. salsa* leaf tissues. The pathway of betaine synthesis is catalyzed by choline monooxygenase converting choline to betaine aldehyde that is subsequently converted into betaine by betaine aldehyde dehydrogenase [37], [75]. Sugars, such as glucose and fructose, are produced from gluconeogenesis and photosynthesis. In plants, sugars are also compatible osmolytes to maintain osmotic balance between plant and environment. Therefore the elevated choline, betaine, fructose and glucose were the indicators of osmotic stress induced by salinity in *S. salsa*.

In realistic situation, *S. salsa* seedlings are exposed to Hg and salinity in a combined manner. After exposure of combined Hg and salinity for 30 days, several metabolites including valine, ethanol, succinate, malonate and phosphocholine were consistently altered with the metabolic responses from Hg-treated *S. salsa* samples, which demonstrated that these metabolic biomarkers induced by Hg were relatively stable under the experimental salinity (NaCl, 500 mM). However, other metabolic responses including isoleucine, leucine, threonine, alanine, choline, malate, fructose and glucose were at control level. It apparently confirmed the antagonistic effects between Hg and salinity. However, asparagine and glycine were uniquely altered in Hg^2+^+salinity-exposed *S. salsa* samples, which suggested that there were synergistic effects between Hg and salinity as well. All these findings indicated that salinity could influence Hg-induced effects in *S. salsa*.

### Conclusions

In this study, the protein changes, metabolic profiles and antioxidant enzyme activities were characterized in the halophyte *Suaeda salsa* with environmentally relevant exposures of Hg^2+^ (20 µg L^−1^), salinity (NaCl, 500 mM) and combined Hg^2+^ (20 µg L^−1^) and salinity (NaCl, 500 mM) for 30 days. Both altered proteins and metabolites of Hg^2+^ exposure were different from those of Hg^2+^+salinity exposure in *S. salsa.* It clearly demonstrated that environmental salinity could influence the chronic effects of mercury in *S. salsa*. Figure 7 draw a broader picture of the effects characterized by both proteomics and metabolomics in *S. salsa* with the exposures of Hg^2+^ and combined Hg^2+^ and salinity. In addition, both proteomic and metabolomic responses indicated the antagonistic and synergistic effects between Hg^2+^ and salinity. Interestingly, six metabolites including valine, ethanol, acetate, succinate, malonate and phosphocholine and one protein, 50S ribosomal protein L12, were consistently regulated in both Hg^2+^ and Hg^2+^+salinity-exposed *S. salsa* samples. These responses could potentially be used as biomarkers of mercury in *S. salsa*, even under an extreme environmental salinity (∼500 mM NaCl). Our results suggest that a combination of proteomics and metabolomics can provide more insightful information of environmental contaminant-induced effects in plants at molecular levels.
